# A deep learning based framework for the classification of multi- class capsule gastroscope image in gastroenterologic diagnosis

**DOI:** 10.3389/fphys.2022.1060591

**Published:** 2022-11-18

**Authors:** Ping Xiao, Yuhang Pan, Feiyue Cai, Haoran Tu, Junru Liu, Xuemei Yang, Huanling Liang, Xueqing Zou, Li Yang, Jueni Duan, Long Xv, Lijuan Feng, Zhenyu Liu, Yun Qian, Yu Meng, Jingfeng Du, Xi Mei, Ting Lou, Xiaoxv Yin, Zhen Tan

**Affiliations:** ^1^ Health Management Center, Shenzhen University General Hospital, Shenzhen University Clinical Medical Academy, Shenzhen University, Shenzhen, China; ^2^ Department of Otorhinolaryngology Head and Neck Surgery, Shenzhen Children’s Hospital, Shenzhen, China; ^3^ Shenzhen Nanshan District General Practice Alliance, Shenzhen, China; ^4^ Group International Division, Shenzhen Senior High School, Shenzhen, China; ^5^ Department of Gastroenterology and Hepatology, Shenzhen University General Hospital, Shenzhen University Clinical Medical Academy, Shenzhen University, Shenzhen, China; ^6^ School of Public Health, Huazhong University of Science and Technology, Wuhan, China

**Keywords:** capsule gastroscope, gastric diseases, diagnosis, deep learning, transfer learning

## Abstract

**Purpose:** The purpose of this paper is to develop a method to automatic classify capsule gastroscope image into three categories to prevent high-risk factors for carcinogenesis, such as atrophic gastritis (AG). The purpose of this research work is to develop a deep learning framework based on transfer learning to classify capsule gastroscope image into three categories: normal gastroscopic image, chronic erosive gastritis images, and ulcer gastric image.

**Method:** In this research work, we proposed deep learning framework based on transfer learning to classify capsule gastroscope image into three categories: normal gastroscopic image, chronic erosive gastritis images, and ulcer gastric image. We used VGG- 16, ResNet-50, and Inception V3 pre-trained models, fine-tuned them and adjust hyperparameters according to our classification problem.

**Results:** A dataset containing 380 images was collected for each capsule gastroscope image category, and divided into training set and test set in a ratio of 70%, and 30% respectively, and then based on the dataset, three methods, including as VGG- 16, ResNet-50, and Inception v3 are used. We achieved highest accuracy of 94.80% by using VGG- 16 to diagnose and classify capsule gastroscopic images into three categories: normal gastroscopic image, chronic erosive gastritis images, and ulcer gastric image. Our proposed approach classified capsule gastroscope image with respectable specificity and accuracy.

**Conclusion:** The primary technique and industry standard for diagnosing and treating numerous stomach problems is gastroscopy. Capsule gastroscope is a new screening tool for gastric diseases. However, a number of elements, including image quality of capsule endoscopy, the doctors’ experience and fatigue, limit its effectiveness. Early identification is necessary for high-risk factors for carcinogenesis, such as atrophic gastritis (AG). Our suggested framework will help prevent incorrect diagnoses brought on by low image quality, individual experience, and inadequate gastroscopy inspection coverage, among other factors. As a result, the suggested approach will raise the standard of gastroscopy. Deep learning has great potential in gastritis image classification for assisting with achieving accurate diagnoses after endoscopic procedures.

## Introduction

Many different stomach disorders, from mild erosive gastritis to advanced cancer, harm the health of a significant portion of the global population. The presence of diffuse or map-like redness in the stomach, together with or without atrophy and mucosal layer erosion, are characteristics of gastritis (Zhang et al., 2022). In Asia, particularly in China, Japan, and Korea, chronic erosive gastritis is a reasonably frequent condition that confounds medical professionals (Goh, 2011). Chronic erosive gastritis, also known as atrophic gastritis, always manifests as a variety of gastrointestinal symptoms and a histological alteration in the stomach mucosa, which lowers the quality of life. Numerous inflammatory lesions in the stomach’s mucous membrane are a defining feature of chronic erosive gastroenteritis. Chronic erosive gastroenteritis is an ulcer-like stomach inflammation marked by many lesions in the mucous lining (Palaniappan, 2013). Their symptoms may include weakness, a loss of appetite, mild nausea, vomiting, and a heavy, burning feeling in the pit of the stomach. Peptic ulcers are produced by erosive gastritis, which can continue damaging the nearing tissues while growing more significantly and broader (White et al., 2022). If the proper diagnosis and treatment are not performed, internal bleeding from severe ulcers may eventually occur, which could cause anemia.

Moreover, an example of peptic ulcer disease is gastric ulcers, which are open sores on the stomach’s lining. Along with the stomach, the intestine can develop ulcers in a section of it. Gastric ulcers may erode our stomach or small intestine’s blood vessel wall. In addition to eating a hole through the lining and becoming infected, ulcers can also grow due to inflammation or scarring, which may prevent food from passing through the digestive tract. Additionally, nodular gastritis, metaplastic gastritis, and open-type atrophic gastritis are linked to stomach cancer, whereas erosive gastritis is associated with obesity and hypoadiponectinemia (Pop et al., 2022). Therefore, during the endoscopic examination, paying attention to the diagnostic signs of gastritis is essential. If patients are examined and treated in the early stage of gastric diseases, the 5-year survival rate can be as high as 90%. However, the early gastric disease detection rate is only about 10%. Without timely diagnosis and proper treatment, long-term inflammation will aggravate the risk of harmful results in a patient’s life (Abbasi-Kangevari et al., 2022). Gastroscopy is the most effective technical tool for identifying and screening numerous gastrointestinal diseases. Gastroscopy allows endoscopists to see stomach lesions by inserting a thin, flexible tube into the stomach. Pathological biopsies of suspected lesions affect the state of the examined part and can confirm a diagnosis. Examining stomach lesions is the preferred method. However, due to exhaustion from lengthy workdays or inexperience, endoscopists may make mistakes during gastroscopy.

To improve gastroscopy diagnosis, numerous imaging techniques have been developed, including 3D imaging, auto-fluorescence imaging (AFI), magnifying endoscopy (ME), and narrow-band imaging (NBI). There is a need for a computer-aided autonomous framework to improve gastroscopy efficiency and quality in daily clinical practice, becoming a “third eye” for endoscopists. Deep learning technology has recently permeated several areas of medical study and has taken center stage in modern science and technology. Deep learning technology can fully utilize vast amounts of data, automatically learn the features in the data, accurately and rapidly support clinicians in diagnosis, and increase medical efficiency. In the field of gastroscopic image analysis, traditional machine learning and deep learning methods have been widely used in disease classification (Lu et al., 2020) and detection (Wong et al., 2012; Wong et al., 2020). Zhang et al. (Zhang et al., 2021) collected gastric images of 308 patients and used the DenseNet model to classify images into atrophic gastric and non-atrophic gastric images. The accuracy of their model combined with serological indicators is 99.25%, with a sensitivity of 96.17%. Qiu et al. (Qiu et al., 2022) classified gastroscopic images into five classes: advanced gastric cancer, early-stage gastric cancer, precancerous lesions, and normal and benign lesions using a convolutional neural network, and the overall accuracy of recognition reached 94.1%. Park et al. (Park et al., 2018) used the transfer learning technique to classify 787 gastric endoscopy images into normal and abnormal classes. After applying the transfer learning technique, the accuracy of the three pre-trained models, including ResNet-50, Inception V3, and VGG- 16, is 98%, 97%, and 98%, respectively. Apart from healthcare, machine learning (Tang et al., 2022) is also used in various fields (Wahid et al., 2021) and also in various domains of life (Zhao et al., 2022) (Ayoub et al., 2022). The authors’ proposed system optimizes treatment and prevents severe kidney stone illness, and they obtained 0.86 sensitivity using a 3D U-Net model. A spherical multi-output Gaussian process may be implemented to model and monitor the 3D surfaces of stones (Hussain et al., 2022). By studying the literature, we observed that the rapid creation of crucial tools for medical diagnostics is being fueled by artificial intelligence (AI), which is quickly becoming a crucial concept in medicine (Wong et al., 2020) (Wei et al., 2017). Deep learning (DL) is now widely employed in medical imaging (Wong et al., 2012) as a critical machine learning method in the field of computer vision (Lu et al., 2020).

Pre-trained deep learning models learned on massive datasets have demonstrated their superiority to conventional approaches as the processing capacity of modern hardware continues to grow. Therefore, from a deep learning perspective, transfer learning can be used to solve the image categorization problem. The study found that the transfer learning technique is used to achieve several cutting-edge achievements in image classification (Simonyan and Zisserman, 2014). We utilized the benefits of pre-trained deep learning models to enhance the diagnosis of capsule gastroscopy images. Our deep learning framework for classifying capsule gastroscopy images into three categories—standard gastroscopic image, chronic erosive gastritis image, and gastric ulcer image—was proposed in this study and is based on transfer learning. We used pre-trained models included VGG- 16, ResNet- 50, and Inception V3 and adjusted their hyper parameters to fit our classification task.

## Materials and Methods

**FIGURE 1:**
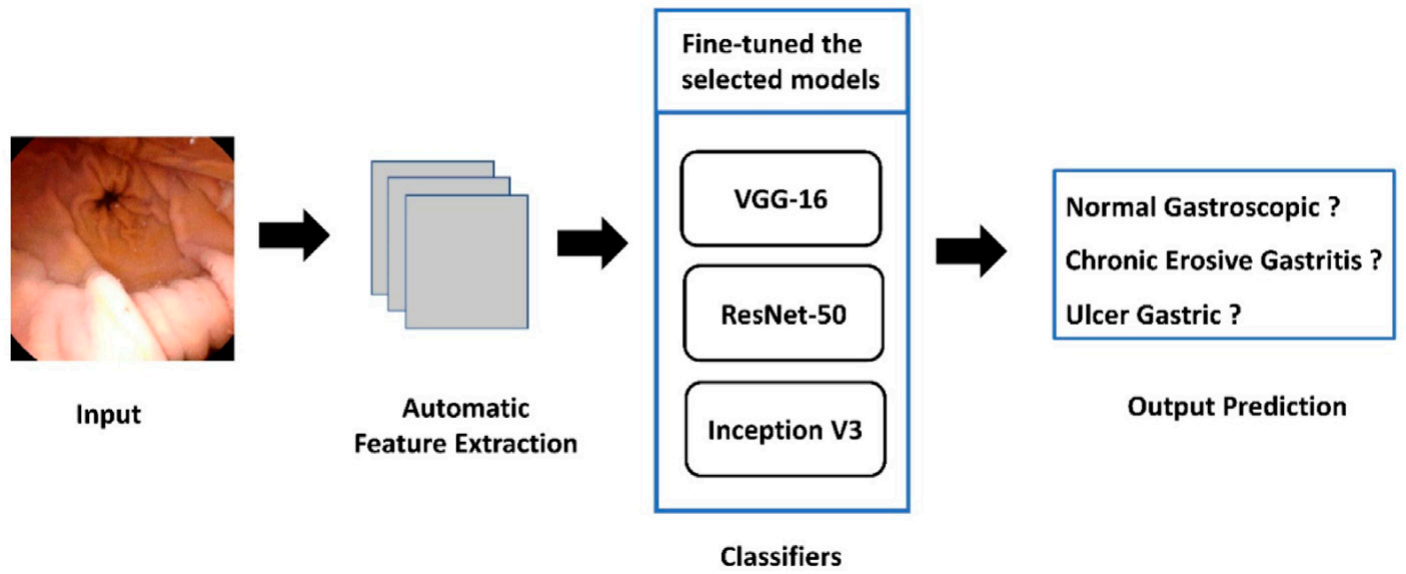
Proposed framework to classify capsule gastroscope images.

To improve capsule gastroscopy image classification, there is a need for a computer-aided autonomous framework to classify capsule gastroscope images into three categories automatically. Deep learning technology has recently permeated several areas of medical study and has taken center stage in modern science and technology (Litjens et al., 2017). Deep learning technology can fully utilize vast amounts of data, automatically learn the features in the data, accurately and rapidly support clinicians in diagnosis, and increase medical efficiency (Ngiam and Khor, 2019). In this research, we proposed a deep learning framework based on transfer learning to classify capsule gastroscopic images into three categories: normal gastroscopic images, chronic erosive gastritis images, and gastric ulcer images. We used VGG- 16, ResNet-50, and Inception V3 pre-trained models, fine-tuned them, and adjusted hyper parameters according to our classification problem by using transfer learning. The proposed framework to address the mentioned research gap is shown in Figure 1. All experiments in this paper are conducted on Intel(R) Celeron(R) CPU N3150 @ 1.60 GHz. The operating system is Windows 64-bit, Python 3.6.6, TensorFlow deep Learning framework 1.8.0, and CUDA 10.1.

### Dataset statistics

**FIGURE 2:**
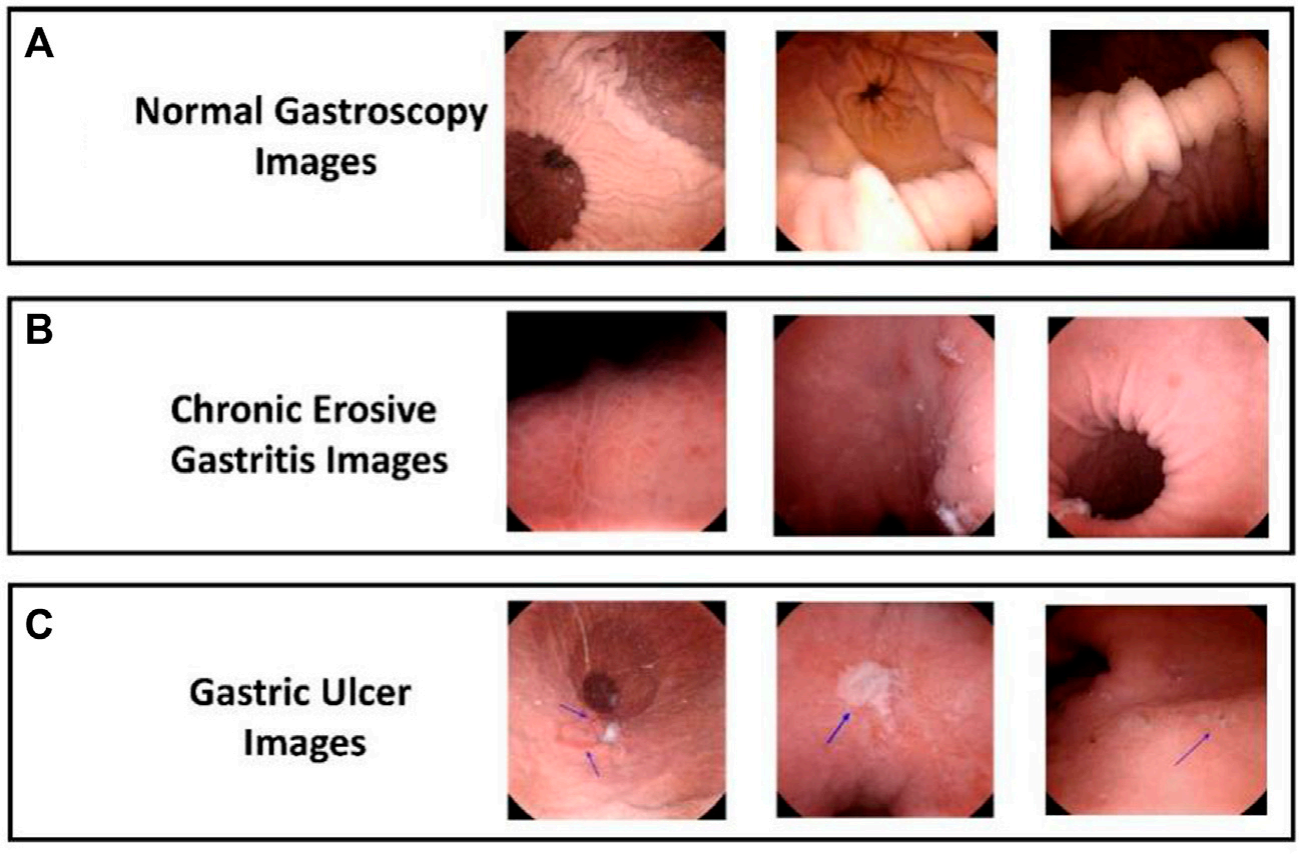
Sample Dataset. **(A)** Representing the normal gastroscopy images, while **(B,C)** representing the images of chronic erosive and gastric ulcer images respectively.

In this study, we gathered 211 patients’ capsule gastroscopic imaging data from Shenzhen University General Hospital, Shenzhen University, China. For each category of capsule gastroscopic images, a total of 1140 lesion samples were randomly selected from 380 different image regions to maintain the balance of disease samples. Then, using a random selection approach, we divide the data in the ratio of 70% and 30% in each type of disease is split into a training set and a test set. Finally, there were 228 test set images and 912 images from the training set. The sample dataset is shown in Figure 2.

Moreover, we also described the statistics of our dataset in table form as shown in Table 1.

**TABLE 1:** Statistics of our dataset in each category.

Type of capsule gastroscopy images	No. of images	No. of lesion samples
Normal Gastroscopy Images	380	1140
Chronic Erosive Gastritis Images	380	1140
Gastric Ulcer Images	380	1140

### Feature extraction

When extracting features, we begin with a pre-trained model and only modify the weights of the last layer, from which we generate predictions. Because we alter the output layer and use the pre-trained CNN as a fixed feature extractor, it is known as feature extraction (Hinterstoisser et al., 2018). Convolution neural networks learn the edge features of the input image and some or all objects—high-level semantic features—successively as the number of convolution steps increases. The convolution layer and full connection layer in the convolution neural network can be utilized to extract the deep features of the image; however, the convolution layer has a multi-dimension, making it challenging to calculate the subsequent dimensionality reduction. However, with a straightforward calculation, the entire connection layer can be viewed as a one-dimensional vector. To represent the deep features of the image, a full connection layer is added before the output layer of the backbone network.

### VGG-16

**FIGURE 3:**
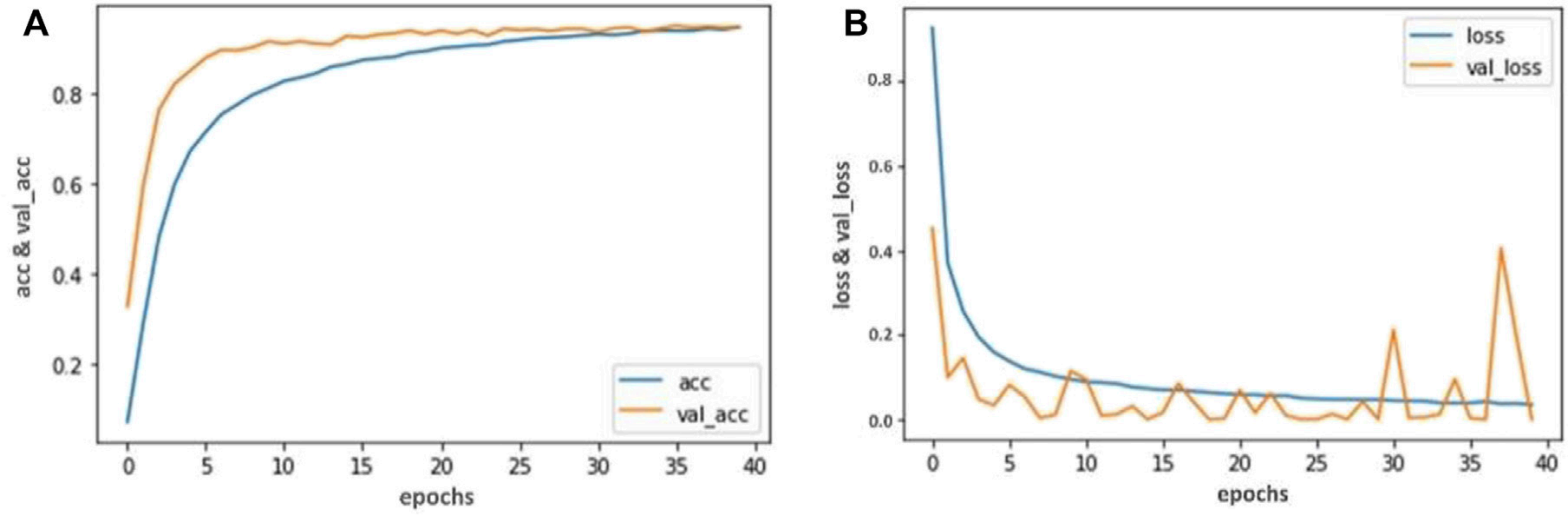
Accuracy and Loss graph using the VGG- 16. **(A)** Representing the training and validation accuracy while **(B)** representing the training and validation loss of VGG- 16 model according to our dataset.

The Visual Geometry Group (VGG) at the University of Oxford developed and trained the convolution neural network model called as VGG- 16 neural network (Guan et al., 2019). The number 16 in VGG- 16 indicates that there are 16 weighted layers. This network has 138 million parameters, which is quite a lot. We used Keras (Poojary and Pai, 2019) to fine-tune the VGG- 16 pre-trained model. We fine-tuned this model according to our dataset to classify capsule gastroscopic images into three categories: normal gastroscopic images, chronic erosive gastritis images, and gastric ulcer images. We reproduced the entire architecture of the VGG- 16 model, excluding the output layer, to produce a new Sequential model. We prevent the output layer from being trained or altered when we feed it our dataset by freezing the weights and other trainable parameters in each layer. Furthermore, we included our new output layer in accordance with our dataset to categorize capsule gastroscopic images into three groups. The complete model architecture and hyper parameter details are shown in Table 2 and Figure 3.

**TABLE 2:** Hyper Parameters details used in VGG-16 model according to our dataset.

Layer (type)	Output shape	Param
Vgg1(Functional)	(None, 7, 7, 512)	14714688
flatten_ 1 (Flatten)	(None, 25088)	0
dense_5 (Dense)	(None, 1024)	25691136
dense_6 (Dense)	(None, 512)	524800
dense_7 (Dense)	(None, 256)	131328
dropout_ 1 (Dropout)	(None, 256)	0
dense_8 (Dense)	(None, 128)	32896
dense_9 (Dense)	(None, 3)	516

### ResNet-50

ResNet-50 is a convolutional neural network with 50 layers (Hussain et al., 2021). A pre-trained version of the network that has been trained on more than a million photos is present in the ImageNet database (Deng et al., 2009). ResNet-50 is a 50-layer residual network in which we endeavor to learn residuals rather than features. In order to solve the problem of the vanishing/exploding gradient, this architecture introduced the concept called Residual network. In this network, we use a technique called skip connections. The skip connection connects activations of a layer to further layers by skipping some layers in between. The residual blocks create an identity mapping to activations earlier in the network to thwart the performance degradation problem associated with deep neural architectures. Another sort of convolutional neural network that it needs input an images size of 224 by 224 and 3 RGB in the ResNet-50. The complete model architecture and hyper parameter details are shown in Table 3.

**TABLE 3:** Hyper Parameters details used in ResNet-50 model according to our dataset.

Layer (type)	Output shape	Param
conv2d (Conv2D)	(None, 26, 26, 28)	784
max_pooling2d	(None, 13, 13, 28)	0
conv2d_ 1 (Conv2D)	(None, 11, 11, 64)	16192
max_pooling2d_ 1	(None, 5, 5, 64)	0
conv2d_2 (Conv2D)	(None, 3, 3, 64)	36928
flatten (Flatten)	(None, 576)	0
dense (Dense)	(None, 640)	369280
dropout (Dropout)	(None, 640)	0
dense_ 1 (Dense)	(None, 264)	169224
dense_2 (Dense)	(None, 64)	16960
dense_3 (Dense)	(None, 3)	260

### Inception V3

Convolutional neural network model Inception-v3 (Wang et al., 2019a) has 48 layers and is also a pre-trained model. A subset of the more than a million images in the ImageNet database was used to train this network. The Google Inception CNN model (Bhatia et al., 2019), which was initially created for the ImageNet Recognition Challenge, is now in its third iteration. Using Inception V3, we were able to reduce the output layer’s dimensions to one, flatten it, and add a sigmoid layer for classification along with a fully connected layer with 1024 hidden units, Relu activation function, and a dropout rate of 0.4. To avoid over-fitting. This method of data augmentation (Shorten and Khoshgoftaar, 2019) operates entirely within memory. The complete model architecture and hyper parameter details are shown in Table 4.

**TABLE 4:** Hyper Parameters details used in Inception V3 model according to our dataset.

Layer (type)	Output shape	Param
inception_v3 (Model)	(None, 8, 8, 2048)	21802784
flatten_ 1 (Flatten)	(None, 131072)	0
activation_95 (Activation)	(None, 131072)	0
dropout_ 1 (Dropout)	(None, 131072)	0
dense_ 1 (Dense)	(None, 1024)	134218752
activation_96 (Activation)	(None, 1024)	0
dropout_2 (Dropout)	(None, 1024)	0
dense_2 (Dense)	(None, 28)	28700
activation_97 (Activation)	(None, 3)	0

One of the best techniques for reducing over fitting is to increase the size of the training dataset (Thanapol et al., 2020). The training images were automatically resized using an augmented image dataset. Our pre-trained deep learning model avoid over-fitting by using dropout layer which is another regularization technique that prevents neural networks from over fitting (Srivastava et al., 2014). Regularization methods like L1 and L2 reduce over fitting by modifying the cost function but on the contrary, the Dropout technique modifies the network itself to prevent the network from over fitting. With the help of data augmentation a lot of similar images can be generated. This helps in increasing the dataset size and thus reduce over fitting. The reason is that, as we add more data, the model is unable to over fit all the samples, and is forced to generalize.

## Result and discussion

Gastroscopy is the primary technique and industry standard for diagnosing and treating numerous stomach problems. The capsule gastroscope is a new screening tool for gastric diseases. However, several elements, including the image quality of capsule endoscopy, the doctor’s experience, and fatigue, limit its effectiveness (Namikawa et al., 2020). Early identification is necessary for high-risk factors for carcinogenesis, such as atrophic gastritis (AG) (González and Agudo, 2012). In this research work, to improve the gastroscopy diagnosis, we proposed a deep learning framework based on transfer learning to classify capsule gastroscope images into three categories: normal gastroscopic images, chronic erosive gastritis images, and gastric ulcer images. 211 patients’ capsule gastroscopic imaging data were gathered from Shenzhen University General Hospital, Shenzhen University, China For each category of capsule gastroscopic images, 1140 lesion samples were randomly selected from 380 distinct image regions to maintain the balance of disease samples. Then, using a random selection approach, we divide the data in the ratio of 70% for training and 30% for the testing set. We used VGG- 16, ResNet-50, and Inception V3 pre-trained models, fine-tuned them, and adjusted hyper parameters according to our classification problem.

**FIGURE 4:**
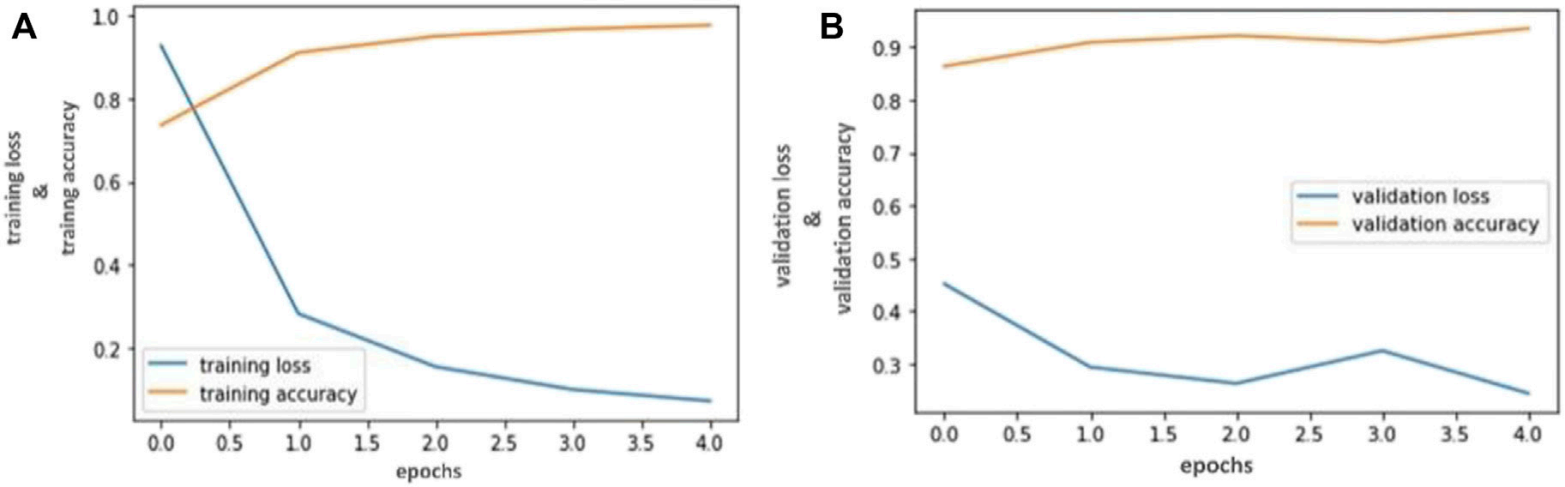
Accuracy and Loss graph using the Inception V3. **(A)** Representing the training loss and training accuracy while **(B)** representing the validation loss and validation accuracy of Inception V3 model according to our dataset.

**FIGURE 5:**
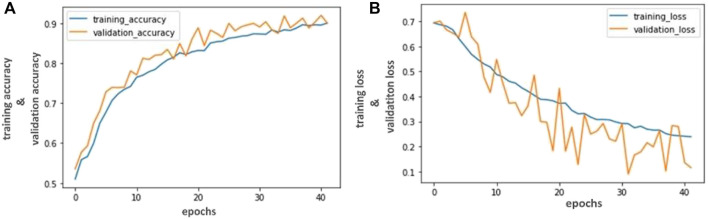
Accuracy and Loss graph using the ResNet-50. **(A)** Representing the training and validation accuracy while **(B)** representing the training and validation loss of ResNet-50 model according to our dataset.

**FIGURE 6:**
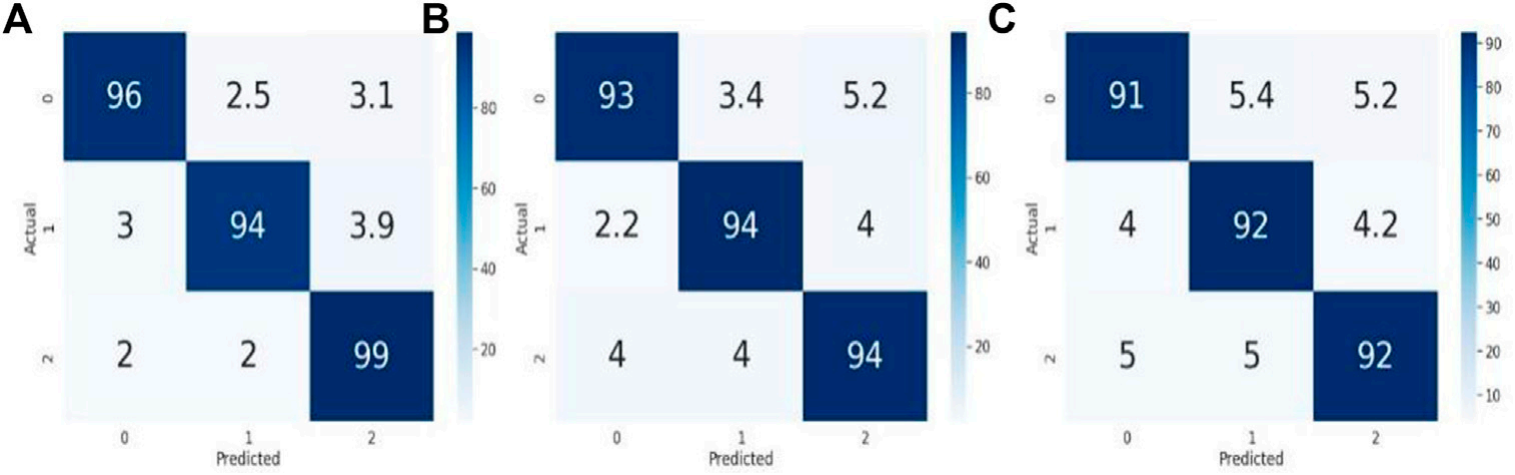
Confusion matrix representation of the performance of our models. **(A)** Representing the performance of VGG- 16, while **(B)** representing the Inception V3 and **(C)** representing the ResNet-50 according to our dataset.

Our trained VGG- 16 model achieved 94.81% accuracy, Inception V3 achieved 92.53% accuracy, and Resnet-50 achieved 90.23% in classifying capsule gastroscopic images into three categories. We assessed our model’s performance using accuracy and loss graphs. Figures 4, 6A reported the training and validation accuracy and (b) training and validation loss using VGG- 16 and ResNet-50 models, respectively, according to our dataset. Similarly, Figure 5 represents the training loss and training accuracy, and (b) represents the validation loss and validation accuracy by using the Inception V3 model in classifying capsule gastroscopic images into three categories.

The most popular method for visualizing the representation of experimentally gathered statistical data confusion matrices is used to solve classification issues in machine learning and deep learning (Wong et al., 2012). Figure 6 represents the performance of three models by using the 3 × 3 confusion matrix. Here (a) illustrates the performance of the VGG- 16 model for classifying capsule gastroscopic images into three categories. Similarly, (b) represents the performance of Inception V3 in terms of the confusion matrix, and (c) illustrates the performance of the ResNet-50 model for classifying capsule gastroscopic images in three categories.

Deep learning technology has recently permeated several areas of medical study and has taken center stage in modern science and technology. Deep learning technology can fully utilize vast amounts of data, automatically learn the features in the data, accurately and rapidly support clinicians in diagnosis, and increase medical efficiency. We used three pre-trained deep learning models to improve the gastroscopy diagnosis, including VGG- 16, Inception V3, and ResNet-50.

We fine-tuned these models according to our dataset to classify capsule gastroscopic images into three categories: normal gastroscopic images, chronic erosive gastritis images, and gastric ulcer images. Our trained VGG- 16 model achieved 94.81% accuracy, Inception V3 achieved 92.53% accuracy, and Resnet-50 achieved 90.23% in classifying capsule gastroscopic images into three categories.

Moreover, we also compared the performance of our proposed approach with previously proposed studies as shown in Table 5.

**TABLE 5:** Comparative accuracy of proposed approach with previously proposed studies.

References study	Approach	Accuracy (%)
Pannu et al. (2020)	Pre-trained Models	93
Wang et al. (2019b)	HaNet	92
Wang et al. (2022)	CNN	89
Our Proposed Approach	Pre-trained Models	94.81

## Conclusion

Deep learning technology has recently permeated several areas of medical study and has taken center stage in modern science and technology. Deep learning technology can fully utilize vast amounts of data, automatically learn the features in the data, accurately and rapidly support clinicians in diagnosis, and increase medical efficiency. Many different stomach disorders, from mild erosive gastritis to advanced cancer, have a negative impact on the health of a significant portion of the global population. Gastroscopy is the most effective technical tool for identifying and screening numerous gastrointestinal diseases. However, due to exhaustion brought on by lengthy workdays or inexperience, endoscopists may make mistakes during gastroscopy. We applied three pre-trained deep learning models, including VGG- 16, Inception V3, and ResNet-50, to enhance the gastroscopy diagnosis. The data was gathered from 211 patients at Shenzhen University Hospital (Shenzhen University Clinical Medical Academy, Shenzhen University, China). To preserve the balance of disease samples, a total of 1140 lesion samples were randomly chosen from 380 different image regions for each category of capsule gastroscopic images. Our trained VGG- 16 model achieved 94.81% accuracy, Inception V3 achieved 92.53% accuracy, and Resnet-50 achieved 90.23% in classifying capsule gastroscopic images into three categories: normal gastroscopic image, chronic erosive gastritis images, and gastric ulcer image. Our suggested framework will help prevent incorrect diagnoses brought on by low image quality, individual experience, and inadequate gastroscopy inspection coverage, among other factors. As a result, the suggested approach will raise the standard of gastroscopy. Investigation of the gastrointestinal functions (Wong et al., 2017) can be enhanced based on variable drug introduction and the reaction may be further analyzed. Advanced bioinformatics algorithms (Li et al., 2017a; Deb et al., 2018) may be utilized to understand the effect of different biochemical environment on gastrointestinal related diseases, which provides valuable information to assist in healthcare (Li et al., 2017b) enhancement.

## Limitation and future work

Due to challenges getting well-annotated data, there is frequently a dearth of training picture collections required for model reconstruction in real-world applications, particularly in the medical industry. Transfer learning, secondary training, fine-tuning, and comparison with the outcomes of self-designed networks were therefore some of the techniques most frequently applied in the works under analysis. Therefore, even though the results of studies have the potential for deep learning associated with different kinds of gastric tissue images, additional studies may need to be carried out clearly and transparently, with database accessibility and reproducibility, in order to develop useful tools that aid health professionals.

## Data Availability

The original contributions presented in the study are included in the article/Supplementary material, further inquiries can be directed to the corresponding author.
